# IL7 in combination with radiotherapy stimulates a memory T-cell response to improve outcomes in HNSCC models

**DOI:** 10.1007/s00262-024-03664-y

**Published:** 2024-03-30

**Authors:** Justin Yu, Jacob Gadwa, Richard B. Ross, Michael Knitz, Laurel B. Darragh, Khalid N. M. Abdelazeem, Jessica Beynor, Brooke Neupert, Alexander Nguyen, Diemmy Nguyen, Nicholas Olimpo, Sophia Corbo, Benjamin Van Court, Angelo D’Alessandro, Anthony Saviola, Sana D. Karam

**Affiliations:** 1 Department of Otolaryngology - Head and Neck Surgery, University of Colorado Anschutz Medical Campus, Aurora, CO 80045 USA; 2 Department of Radiation Oncology, University of Colorado Anschutz Medical Campus, Aurora, CO 80045 USA; 3 Department of Immunology and Microbiology, University of Colorado Anschutz Medical Campus, Aurora, CO 80045 USA; 4 Radiation Biology Research Department, National Center for Radiation Research and Technology, Egyptian Atomic Energy Authority, Cairo, Egypt; 5 Department of Biochemistry and Molecular Genetics, University of Colorado Anschutz Medical Campus, Aurora, CO 80045 USA

**Keywords:** Cancer immunology, Immunotherapy, Interleukin-7, Radioimmunotherapy, Memory T cells

## Abstract

Clinically approved head and neck squamous cell carcinoma (HNSCC) immunotherapies manipulate the immune checkpoint blockade (ICB) axis but have had limited success outside of recurrent/metastatic disease. Interleukin-7 (IL7) has been shown to be essential for effector T-cell survival, activation, and proliferation. Here, we show that IL7 in combination with radiotherapy (RT) is effective in activating CD8 + T-cells for reducing tumor growth. Our studies were conducted using both human papillomavirus related and unrelated orthotopic HNSCC murine models. Immune populations from the tumor, draining lymph nodes, and blood were compared between treatment groups and controls using flow cytometry, proteomics, immunofluorescence staining, and RNA sequencing. Treatment with RT and IL7 (RT + IL7) resulted in significant tumor growth reduction, high CD8 T-cell tumor infiltration, and increased proliferation of T-cell progenitors in the bone marrow. IL7 also expanded a memory-like subpopulation of CD8 T-cells. These results indicate that IL7 in combination with RT can serve as an effective immunotherapy strategy outside of the conventional ICB axis to drive the antitumor activity of CD8 T-cells.

**Supplementary Information:**

The online version contains supplementary material available at 10.1007/s00262-024-03664-y.

## Introduction

Head and neck squamous cell carcinoma (HNSCC) is estimated to be the sixth most common cancer worldwide [1, 2]. The incidence of HNSCC continues to rise and is expected to increase by 30% by 2030 [1–3], with mortality rates reported between 40 and 50% [4]. HNSCC is clinically divided into two major divisions: human papillomavirus (HPV) related (HPV+) and unrelated (HPV−). Traditionally, HPV− HNSCC development is associated with exposure to tobacco-related carcinogens and/or excessive alcohol use [5, 6]. HPV+ HNSCC incidence has increased due to higher exposure to oncogenic strains of the virus and now comprises a majority of diagnosed oropharyngeal HNSCCs [7, 8].

The advent of immunotherapy has played a large role in revolutionizing the treatment of various cancers. In many cancer subtypes, immunotherapy in combination with radiotherapy (RT) has improved response rates and lengthened overall survival (OS) for patients [9]. Most approved FDA immunotherapies focus on altering immune checkpoints, most frequently targeting the programmed cell death protein 1 (PD1) and programmed death-ligand 1 (PD-L1) axis to prevent T-cell exhaustion [10–13]. These therapies work to stimulate tumor infiltrating lymphocytes (TILs) to continually mount an effector response against cancer cells. However, in the field of HNSCC, the benefit of immunotherapies has mainly been limited to recurrent and metastatic disease [14, 15]. Several studies, including Javelin Head Neck 100, Reach-Gortec, Keynote-412, and HN-004, have incorporated immune checkpoint blockade (ICB) to chemoradiotherapy or cetuximab-RT but have not shown significant oncologic improvement in locally advanced HNSCC (NCT 02952586, NCT02952586, NCT03040999, NCT03258554) [16–18]. As a result, there is a need for development of immunotherapies outside of the PD1/PD-L1 pathway.

Interleukin-7 (IL7) has long been theorized as a potential therapeutic agent in cancer treatment. IL7 is known to play an essential role in multiple T-cell pathways, including maturation, survival, maintenance, and proliferation [19]. Moreover, IL7 has been shown to play an important role in the development and preservation of memory immune cell function. Previous work has demonstrated that IL7 can induce naive T-cells to differentiate into a memory phenotype [20, 21], and promote the proliferation and activity of effector T-cells against cancer [22–24]. In addition, suppressive regulatory T-cells (Tregs) have been shown to express relatively low levels of the IL7 receptor (IL7R) [25]. Taken together, these findings suggest that IL7 may be a prime target for development as a cancer immunotherapy.

In this work, we investigate the antitumor properties of IL7 in both HPV+ and HPV− murine models. We show that IL7 in conjunction with RT works to decrease tumor growth through stimulation and maintenance of a memory T-cell phenotype, notably without a parallel increase in Treg influx into the tumor microenvironment (TME). We demonstrate that the antitumor effect is amplified when IL7 is paired with Treg depletion. In addition to directly acting on the tumor, IL7 exerts effects on lymphopoiesis by stimulating T-cell precursor development in the bone marrow. We believe this work is highly translational and provides a therapeutic drug target for HNSCC that is outside of the conventional ICB axis.

## Methods

### Cell lines

mEER (HPV+) and LY2 (HPV−) murine squamous cell carcinoma cells lines were used for in vivo studies. The mEER cell line was obtained from Dr. John Lee (Sanford Health, Sioux Falls, SD), and the LY2 cell line was from Dr. Nadarajah Vigneswaran (University of Texas Health Science Center, Houston, TX). The mEER cells were cultured in DMEM-F12 with 10% FBS, 1% primocin/fungin, 0.5 μg/mL of hydrocortisone, 5 μg/mL of insulin, 1.5 ng/mL of tri-iodo-thyonine, 5 μg/mL of transferrin, 5 ng/mL of EGF, and 10 ng/mL of cholera toxin. The LY2 cells were cultured in DMEM-F12 with 10% FBS and 1% primocin/fungin.

### Mouse models

C57BL/6 and BALB/c female mice were obtained from Jackson Laboratory (Bar Harbor, Maine, USA) and Charles River (Wilmington, MA, USA) and were used for in vivo studies. Mice were between 6 and 12 weeks and age matched between experimental groups. Mice were housed under pathogen free conditions with a maximum of 5 per cage. All protocols for animal models were approved by the Institutional Animal Care and Use Committee (IACUC) of the University of Colorado Anschutz Medical Campus.

### Tumor studies

Murine mEER and LY2 cells were orthotopically implanted into the buccal mucosa as previously described [26]. After implantation, mice were randomized into different treatment groups. Treatment was initiated when tumor volume was approximately between 150 and 250 mm^3^. Tumor size was measured at least twice a week using digital calipers, and volume was computed using the formula *V* = (*A* × *B*^2^)/2, where *A* was defined as the longer diameter, and *B* is defined as the shorter diameter of the tumor. For both cell lines, 1 million cells were implanted into the buccal mucosa. Primary tumor, lymph node, and serum were harvested at the time of sacrifice. Samples for flow cytometry were collected on post-implantation days 15 and 22; samples for proteomics were collected at day 15.

### Human patient samples

Human samples were obtained from HNSCC patients from a completed phase I/Ib clinical trial (NTC:03635164) at the University of Colorado Anschutz Medical Campus [27]. Tumor tissue collected from the initial biopsy and time of surgery was used for RNA sequencing. Blood taken from patients was processed to collect PBMCs and plasma. Written informed consent was obtained from all participants prior to performing any procedures.

### TCGA/Tisch data sets

The HNSCC dataset was downloaded from TCGA, and gene expression was extracted for *IL7*, *SELL*, and *KLRG1*. Patients were stratified by median expression into either quartiles or halves. *IL7* expression was correlated with 2 year progression free interval (PFI), and *SELL* and *KLRG1* were correlated with overall survival (OS). Kaplan–Meier curves were drawn, and statistics were computed using the log-rank test.

Comparative gene expression and annotated cell maps from human single cell RNA sequencing datasets were generated using the Tumor Immune Single-cell Hub (TISCH) database [28]. Cillo et al. (GEO: GSE139324) [29] was a head and neck single cell RNA sequencing dataset included in the analysis.

### Antibodies and drugs

Recombinant human IL7 was provided by the Biological Resources Branch (BRB) National Cancer Institute (NCI). This was given at a concentration of 0.5 mg/kg through an intraperitoneal (i.p.) injection three times a week for two weeks, starting at the time of irradiation. αCD25 was provided in collaboration with Roche Pharmaceuticals. αCD25 was given at a concentration of 3 mg/kg via i.p. injection, once a week starting at the time of irradiation.

### Irradiation

Irradiation was performed using the Precision (Madison, CT) PXi-225Cx image guided irradiator at 225 kV, 20 mA with a 0.3 mm Cu filter. Mice were anesthetized with vaporized isoflurane and placed in the prone position, and RT was delivered at a dose rate of 5.6 Gy/min. Dose rates are checked monthly using an ionization chamber, and continuous fluoroscopy prior to irradiation was utilized to determine accurate positioning of mice. The buccal region of the mouse was specifically targeted using this manual fluoroscopy, and caution was taken to avoid excess irradiation to the neck and cervical lymph nodes.

### Flow cytometry

Tumor, blood, and tumor draining lymph nodes were harvested and processed for flow cytometric analysis. 4–5 animals were used per group. Tumor tissue was minced and incubated in Collagenase III (Worthington) for 30 min at 37 °C. After incubation, tissue was passed through a 70 μm nylon cell strainer to produce a single cell suspension. Draining LNs were identified at time of dissection as the most enlarged node adjacent to the tumor. After harvesting, these were similarly processed by mechanical separation into single cell suspension. After centrifugation, red blood cells were lysed using RBC lysis buffer (Invitrogen), using HBSS to neutralize the lysis buffer. Bone marrow was harvested using the femur and tibia. These bones were placed into small 0.6 mL microtubes with a hole punctured in the bottom using an 18-gage needle. These were then stacked into 1.5 mL microtubes and spun at 10,000 rpm for 5 min. Bone marrow was harvested from pellets that had collected in the bottom of the larger microtube.

Cells were transferred into 24 well plates and incubated with monensin and brefeldin to prevent release of cytokines and stimulated with PMA/ionomycin cocktail for 4 h at 37 °C. Following incubation, cells were incubated in FC block (CD16/CD32 antibody, Tonbo bioscience) for 15 min at 4 °C. Cells were then incubated in Live/Dead Fixable Aqua Viability Stain Kit (Invitrogen) in the dark for 20 min at 4 °C. Cells were then stained for surface markers and incubated for 20 min at 4 °C. For analysis of immune cells, the following antibodies were used:

PerCP–CD45 (clone: 30-F11, Biolegend), eF450–CD3 (clone: 17A2, Invitrogen), BUV496–CD4 (clone: GK1.5, BD Biosciences), APC-eF780–CD8 (clone: 53–6.7, Invitrogen), BV570-CD44 (clone: IM7, Biolegend), BUV750–CD25 (clone: PC61, Biolegend), BV785–CD62L (clone: MEL-14, Biolegend), APC-Fire810–PD1 (clone: 29F.1A12, Biolegend), BUV615–CD11c (clone: N418, Invitrogen), PE/Dazzle 594–CCR7 (clone 4B12, Biolegend), BUV661–CD11b (clone: M1/70, BD Biosciences), BUV563–CD19 (clone: 1D3, Invitrogen), PE-Cy5–Ki67 (clone: SolA15, Invitrogen), AF532–FoxP3 (clone: FjK-16s, Invitrogen), BV605–IL2 (clone: MQ1-17H12, BD Bioscience), BUV737–IFNγ (clone: XMG1.2, BD Biosciences), APC–IL10 (clone: 554,468, BD Biosciences), BV421–Tbet (clone: 4B10, Biolegend), PE-Cy7–GRMB (clone NGZB, Invitrogen),

PerCP–Cy5.5-KLRG1 (clone: 2F1, BD Biosciences), BV711–CCR8 (clone: SA214G2, Biolegend), AF647–CD127 (clone: A7R34, Biolegend), AF488–TCF1 (clone: IC8224G, R&D Systems), PE–TOX (clone: TXRX10, Invitrogen), BV650–CXCR3 (clone: CXCR3-173, Biolegend), BUV395–CD103 (clone: 2E7, BD Biosciences), AF700–MCHII (clone: M5/114.15.2, Biolegend).

For analysis of bone marrow, the following antibodies were used:

Lin-FITC (clone: 145-2C11, RB6-8C5, RA3-6B2, Ter-119, M1/70, Biolegend), Sca1-PerCP (clone: D7, Biolegend), cKit-APC (clone: 2B8, Invitrogen), CD34-PeCy7 (clone: HM34, Biolegend), CD48-APC-Fire750 (clone: HM48-1, Biolegend), CD135-BV421 (clone: A2F10.1, Invitrogen), CD150-PE (clone: 9D1, Invitrogen), CD25-BV711 (clone: PC61, Biolegend), CD44-BV570 (clone: IM7, Biolegend), B220-BV750 (clone: RA3-6B2, Biolegend).

After surface staining, cells were fixed and permeabilized using the FoxP3 perm/fix kit (Invitrogen) overnight. Following incubation, cells were stained for intracellular markers and incubated for 30 min at 4 °C. Samples were then run on a Cytek Aurora spectral cytometer at the University of Colorado Diabetes Research Center Flow Cytometry Core. Fluorescence minus one (FMO) controls were used to determine gating strategy. Flowjo (Ashland, OR) software was used for gating and data analysis. Percentages of cell subtypes, such as CD4+ and CD8+ , were used to account for differences in tumor size.

### Immunofluorescence staining

Tumors were harvested at time of killing and fixed in 10% buffered formalin. Tissue was submitted to University of Colorado Denver Morphology and Phenotyping Core, where slides were cut and mounted. Tissue was mounted using every 3rd cut. Primary antibodies against CD8 and CD127 were from rat and rabbit, respectively. Secondary antibodies were conjugated with a fluorophore and targeted against these organisms. Alexa fluor 647 goat anti-rabbit and Alexa fluor 488 donkey anti-rat were used.

### Proteomics

Tumor and lymph nodes were collected from tumor-bearing mice for bulk proteomics analysis by the University of Colorado Mass Spectrometry Shared Resource as described previously in our publications [30]. Approximately 500 μg of lyophilized tissue was resuspended in 8 M urea, 0.1 M Tris (pH 8.5), 5 mM TCEP (tris(2-carboxyethyl)phosphine) and incubated with constant agitation (1400 rpm) for 2 h at 37 °C. Samples were then alkylated with 50 mM 2-chloroacetamide for 30 min in the dark at room temperature. The solutions were diluted with four volumes of 100 mM Tris–HCl (pH 8.5) before digestion with Lys-C (1:100) (PierceTM) for 2 h at 37 °C with constant shaking (1400 rpm). Samples were then digested overnight with trypsin (1:100) followed by a second treatment of trypsin for 2 h. Both steps were carried out with continuous agitation (1400 rpm) at 37 °C. Following trypsin digestion, samples were acidified with formic acid to a final concentration of 1%, centrifuged at 16,000 × g for 5 min at room temperature, and the supernatant was collected. Aliquots containing 10 μg of digested peptides were purified using PierceTM C18 Spin Tips (Thermo Scientific) according to the manufacturer's protocol, dried in a vacuum centrifuge, and resuspended in 0.1% FA in mass spectrometry-grade water.

Liquid chromatography–tandem mass spectrometry (LC–MS/MS) was performed using an Easy nLC 1200 instrument coupled to an Orbitrap Fusion Lumos Tribrid mass spectrometer (ThermoFisher Scientific). Proteolytic peptides were separated on a C18 column (100 μM inner diameter × 20 cm) packed in-house with 2.7 μm Cortecs C18 resin. The flow rate was set to 0.4 μL/min, and the column was developed with a linear gradient of 0.1% formic acid in ddH2O (solution A) and 0.1% formic acid in 80% ACN (solution B) at 6% B for 3 min, followed by 6–42% B for 102 min, 42–60% B for 5 min, 60–95% B for 1 min, isocratic at 95% B for 9 min. The Orbitrap Fusion Lumos was set to 120 K resolution, and the top N precursor ions in a 3-s cycle time (within a scan range of 300–1800 m/z) were subjected to high-energy collision dissociation (HCD) with 30% collision energy for peptide sequencing using a 15 K resolution setting. MS/MS was performed on the most abundant precursors exhibiting a charge state from 2 to 7 and an intensity threshold of 2 × 104. Dynamic exclusion was set to 45 s with a 10 ppm mass tolerance.

### RNA sequencing

Tumors were harvested from mice for RNA extraction with an RNA miniprep kit (Zymo Research, Irvine, CA). Sequencing and library prep were performed by The Genomics and Microarray Shared Resource at University of Colorado Denver Cancer Center. mRNA was isolated for library prep and sequencing was performed on an Illumina NovaSEQ 6000 with 2 × 150 paired end (PE) reads at a depth of 40 million PE reads per sample. Illumina adapters and the first 12 base pairs (bp) of each read were trimmed using BBDuk (BBMap, Bushnell B., sourceforge.net/projects/bbmap/), and reads < 50 bp post trimming were discarded. Reads were aligned and quantified using STAR (2.6.0a) against the Ensembl mouse transcriptome (mg38.p6 genome [release 96]). Genes with low expression were removed if the mean raw count was less than 1 or if mean counts per million (CPM) was less than 1.

### Quantification and statistical analysis

All statistical analyses were processed using GraphPad Prism v9 (Boston, MA). Statistical analysis with multiple groups was completed using one-way analysis of variance (one-way ANOVA). Pairwise comparisons were performed using Fisher’s least significant difference test. Unpaired *t* tests were used for comparisons between only two groups. Kaplan–Meier curves were used for survival analysis, and p values computed using the log-rank test. Data are represented as the mean with error bars signifying the standard error of the mean (SEM) unless otherwise indicated. Statistical significance is indicated as *p < 0.05, **p < 0.01, ***p < 0.001, ****p < 0.0001.

## Results

### Expression of IL7, IL7R, and memory phenotypes in human data

**Fig. 1:**
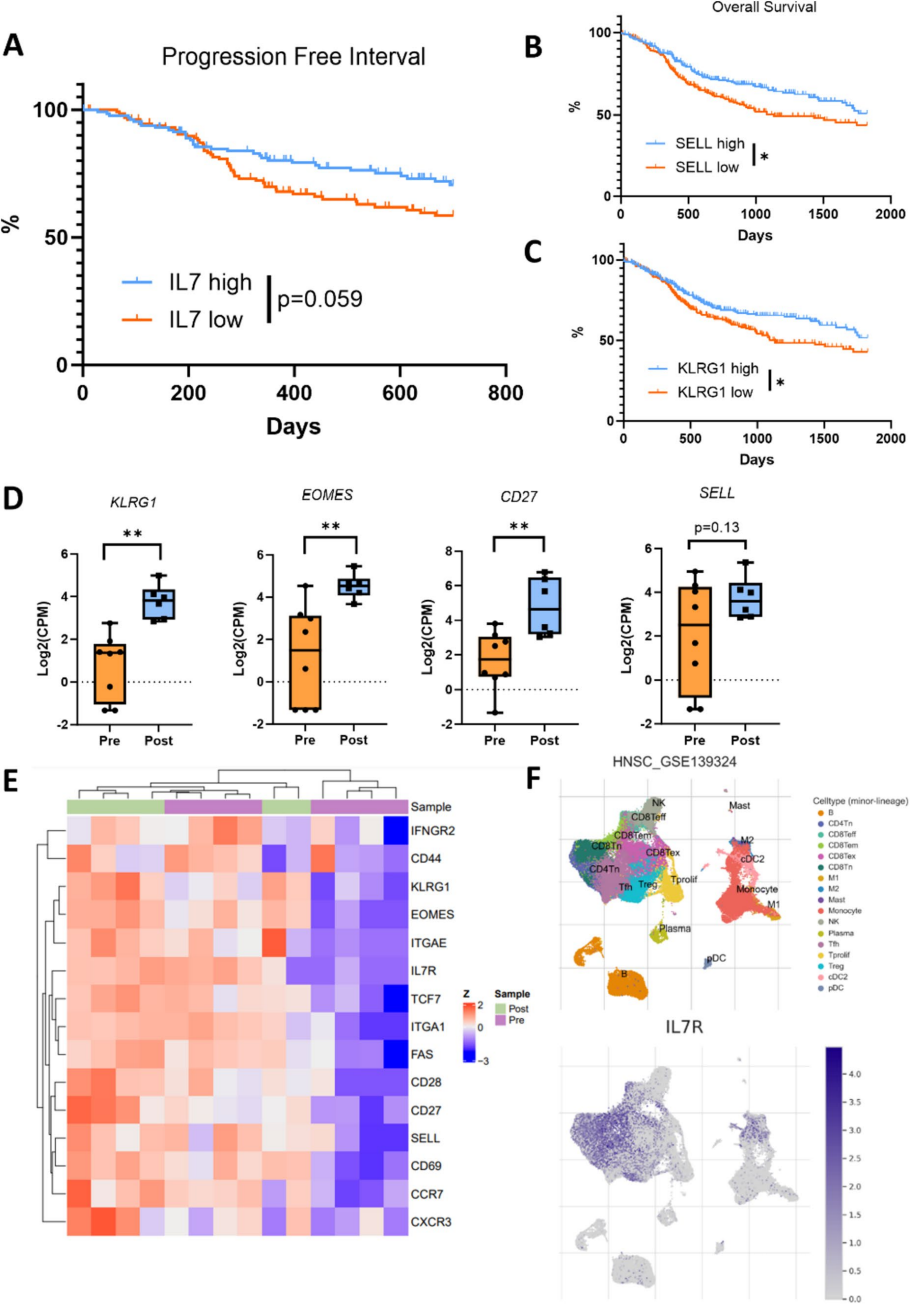
Expression of IL7, IL7R, and memory markers in human data. **A** Progression free interval (PFI) of patients in the TCGA, stratified by high and low expression of IL7. **B**–**C** Overall survival (OS) of patients in the TCGA, stratified by high and low SELL and KLRG1 expression. **D**–**E** Differentially expressed genes related to lymphocyte memory in human responders to radioimmunotherapy. RNA sequencing performed pre- and post-treatment. Corresponding heat map is normalized by *z*-scores of gene expression. **F** Single cell RNA sequencing data from the TISCH dataset clustered into immune subpopulations. IL7R expression queried based on cell subtype. Statistics were assessed by the log-rank test (**A**–**C**) and the moderated t test from the limma R package (**D**). *p < 0.05, **p < 0.01

In support of our study rationale, we queried The Cancer Genome Atlas (TCGA) to evaluate the role of IL7 and other memory T-cell markers on human patient outcomes in HNSCC. When stratified into high and low IL7 expression based on the upper and lower quartiles, it was found that high IL7 gene expression trended toward prolonged progression free interval (PFI) over a 2 year period (Fig. 1A) (HR 0.65, 95% CI [0.423, 1.01], *p* = 0.059). Memory markers were then examined in the TCGA to determine if cellular memory phenotypes play a role in extending survival. Patients were stratified into high or low categories based on median gene expression for the memory markers *SELL,* the gene that encodes CD62L, and *KLRG1*. Both were associated with increased 5 year overall survival (OS) (Fig. 1B, C) (*SELL* HR 0.70, 95% CI [0.532, 0.927], *p* = 0.0127; *KLRG1* HR 0.73, 95% CI [0.552, 0.959], *p* = 0.0245).

The role of memory phenotypes in improving patient outcomes was further assessed by analyzing a recently completed phase I/Ib clinical trial using neoadjuvant stereotactic body RT (SBRT) with durvalumab (anti-PD-L1) [27]. RNA sequencing (RNAseq) of tumors was performed on patients who were deemed histologic “responders” both pre-treatment (*n* = 8) and post-treatment (*n* = 6). In this group, there were post-treatment increases in *SELL* (*p* = 0.13)*, KLRG1* (*p* < 0.005)*, EOMES* (*p* < 0.005)*,* and *CD27* (*p* < 0.005) expression, which are all important markers in development of cellular memory (Fig. 1D, E).

To understand IL7 therapeutic targets and potential IL7R cellular partners, we examined the expression profile of IL7 using a publicly available human single-cell RNAseq dataset. This dataset was generated using the Tumor Immune Single Cell Hub (TISCH), and IL7R expression was superimposed on major cell lineages. IL7R was found to be expressed mainly on CD8 and CD4 T-cells, particularly on naive (Tn) and effector memory (Tem) subpopulations, implicating these cell types as important IL7 targets. Scattered expression was noted across other cell types, including Tregs and T-follicular helper cells (Tfh) (Fig. 1F). Taken together, these data suggest that IL7 acts on effector T-cells, with a focus on memory T-cells, and the development of a memory immune cell phenotype is associated with improved patient outcomes.

### Decreased tumor growth associated with IL7 administration in murine HNSCC

**Fig. 2:**
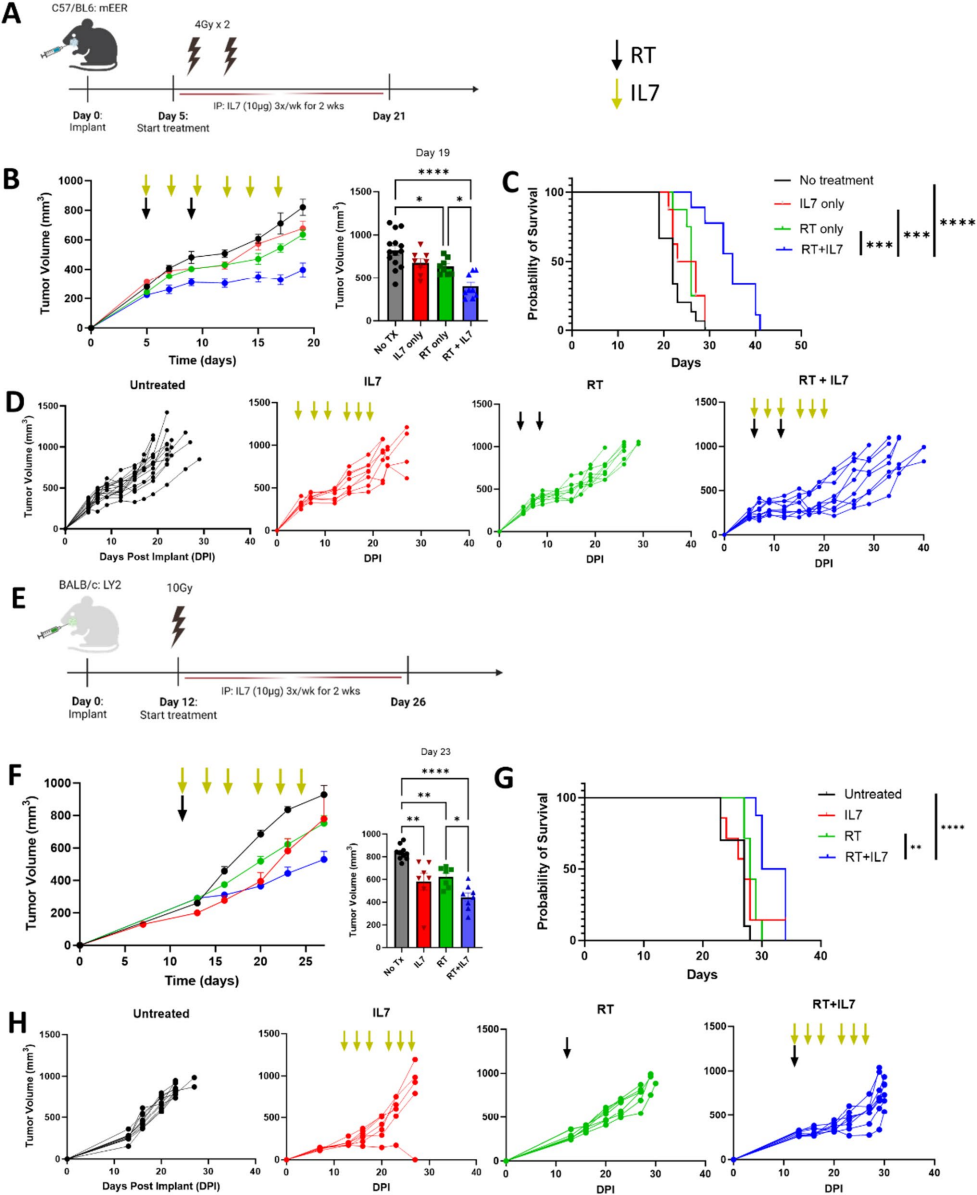
IL7 decreases tumor growth in murine HNSCC models. **A** Schematic of experimental design for the orthotopic mEER HPV+ murine model in C57BL/6 mice. Initiation of treatment started on day 5 with two fractions of 4 Gy RT combined with 2 weeks of IL7 i.p. injections. **B** Tumor growth curves with untreated (*n* = 15), RT (*n* = 8), IL7 (*n* = 9), and RT + IL7 (*n* = 9) cohorts with timing of treatment indicated by yellow and black arrows. Experiments were repeated and pooled. **C** Kaplan–Meier survival plot for the mEER model stratified by treatment. **D** Individual growth curves for each treatment are shown. **E** Experimental design for the orthotopic LY2 HPV− murine model in BALB/c mice. Initiation of treatment starting at day 12 after implantation. One dose of 10 Gy RT was given with 2 weeks of i.p. IL7 injections. **F** Tumor growth curves for untreated (*n* = 10), IL7 (*n* = 7), RT (*n* = 8), and RT + IL7 (*n* = 8) groups with timing of treatments indicated by yellow and black arrows. Experiment was repeated and pooled. **G** Kaplan–Meier survival plot for the LY2 model stratified by treatment. **H** Growth of individual LY2 tumors shown for each group. Statistics were determined using one-way ANOVA testing (**B**, **F**) and log-rank testing (**C**, **G**). The mean ± SEM is shown (**B**, **F**). *p < 0.05, **p < 0.01, ***p < 0.001, ****p < 0.0001

As IL7 was linked to improved patient outcomes and T-cell memory development, we hypothesized that the addition of IL7 into the TME would result in reduced tumor growth, which was studied using mouse orthotopic HNSCC models. This was first performed in C57BL/6 mice with the mEER cancer line, an E6-E7 driven murine cancer line (Fig. 2A). Coadministration of RT and IL7 (RT + IL7) was found to significantly reduce tumor growth (Fig. 2B) and prolong overall survival compared to RT or IL7 alone (Fig. 2C). No statistically significant increase in tumor reduction was found when comparing IL7 alone with the untreated group, demonstrating the importance of RT even in hot, virally mediated tumors (Fig. 2D). In a second mouse model, the LY2 HPV− cancer cell line was implanted into BALB/c mice (Fig. 2E). Similar to the mEER model, the RT + IL7 cohort had reduced tumor growth and prolonged survival outcomes compared to the other groups (Fig. 2F–H).

### IL7 drives activation of lymphocytes in the LN within days post treatment

**Fig. 3:**
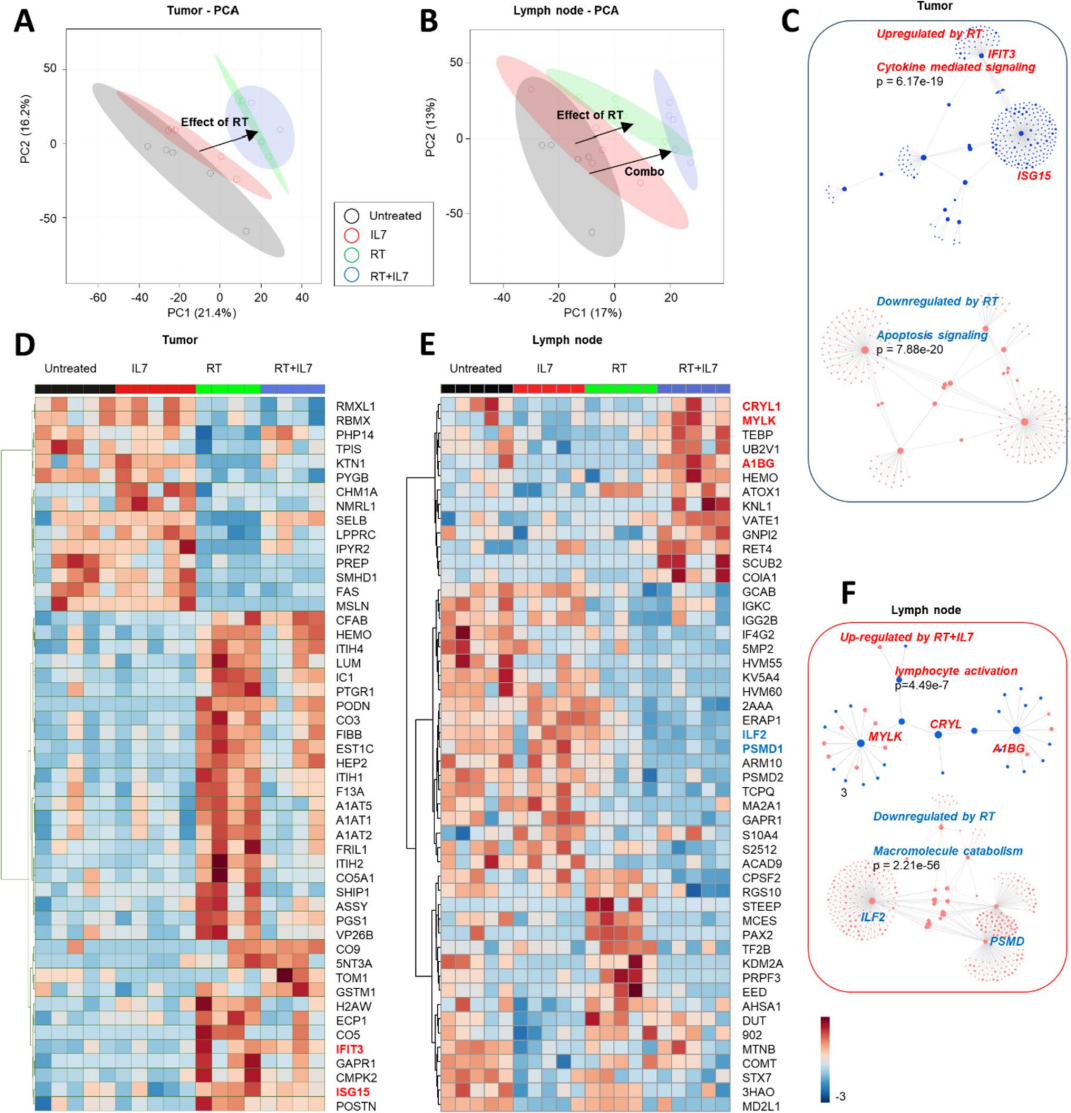
Proteomics shows early activation of lymphocytes occurs in draining lymph node. **A**–**B** Principal component analysis (PCA) of tumor proteomics (**A**) and lymph node proteomics (**B**) with untreated, IL7, RT, and RT + IL7 groups (*n* = 5 per group). Black arrows indicate the effect of each treatment. **C**, **F** Network analysis of pathways that are up- and down-regulated in the tumor (**C**) and the lymph node (**F**). Interferon-related gene products IFIT3 and ISG15 were among the most significantly up-regulated nodes in the tumor, and proteins involved in lymphocyte activation, such as MYLK, CRYL and A1BG, were upregulated in the node. **D**–**E** Heatmap of differentially expressed proteins in the tumor (**D**) and lymph node (**E**) between the 4 treatment groups. Heatmap coloring based on normalized *z*-scores

The effect experiment with the mEER cell line was repeated, and tumor and LN were harvested on day 15 to understand how early protein expression was affected. Tumor proteomics showed a strong effect of RT, alone or in combination with IL7, as represented by unsupervised principal component analysis (PCA) (Fig. 3A). Hierarchical clustering analysis of the top 50 proteins impacted by either treatment or the combo (Fig. 3C) identified a significant RT-dependent upregulation of proteins involved in cytokine-mediated signaling and antiviral responses (*p* < 0.0001), with interferon-related gene products IFIT3 and ISG15 ranking among the most significantly up-regulated nodes in this network. Of note, RT—especially when alone and less so when in combination with IL7—was associated with signatures of extracellular matrix remodeling through inflammatory and pseudo-coagulation processes, as suggested by the up-regulation of proteins involved in this pathway, including complement components (CFAB, CO3, CO5A1, CO9), serine protease inhibitors (ITIH1, ITIH4, A1AT1, A1AT2, A1AT5), fibrinogen chains (FIBB), heme-binding proteins (hemopexin—HEMO) and coagulation factors with cross-linking transglutaminase activity (Fig. 3D). RT was also associated with downregulation of apoptosis signaling (*p* < 0.0001). Flow cytometry at this time demonstrated uniform CD8 T-cell influx into the TME, with an increase in Ki-67 expression in the RT and RT + IL7 groups (Supp Fig. 1A, B). Proteomics pathway analysis demonstrated an upregulation of metabolic pathways in RT + IL7 and RT groups including oxidative phosphorylation, cellular respiration, and ATP synthesis (Supp Fig. 1C–E).

Proteomics of the draining LN demonstrated a unique signature of RT + IL7 as inferred from PCA (Fig. 3B). While RT alone had the strongest effect (Fig. 3E), especially with respect to downregulation of macromolecule catabolism and mRNA metabolic processes (*p* < 0.0001), the combo RT + IL7 therapy causes a significant upregulation of proteins involved in lymphocyte activation (*p* < 0.0001), with MYLK, CRYL and A1BG ranking as the clustering nodes with the highest degree in this network (Fig. 3F). Along the same lines, flow cytometry demonstrated increased CD8 and CD4 T-cell activation via heightened expression of CD44 and Ki-67 in the LN (Supp Fig. 2A–C). In summary, these data taken together indicate that lymphocyte activation occurs early in the draining LN, days after treatment initiation.

### Increased CD8 immune influx into TME over time

**Fig. 4:**
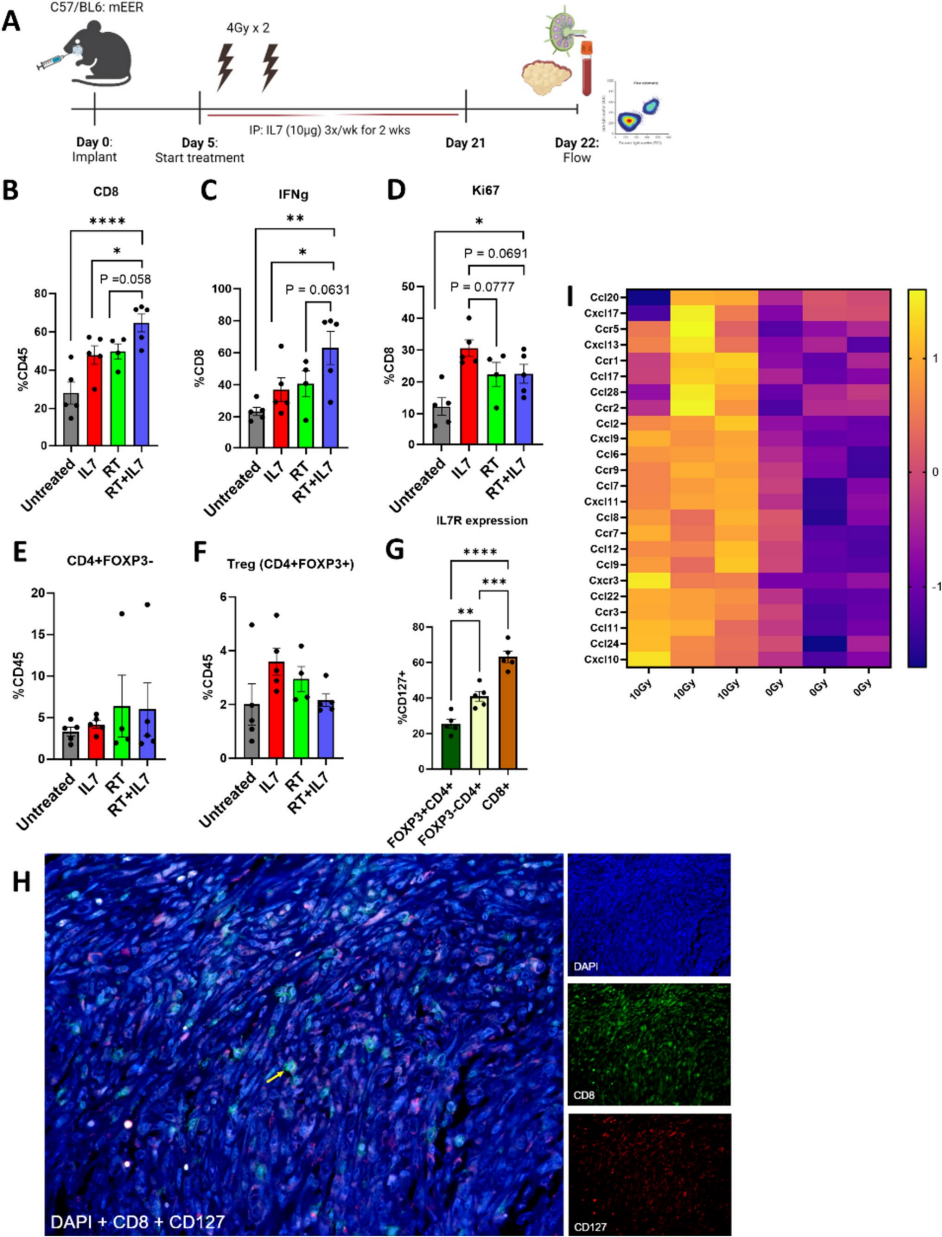
Increased CD8 T-cell influx and activity in TME with RT + IL7 treatment. **A** Experimental schematic showing timepoints of treatment. Mice were treated with two fractions of 4 Gy RT and two weeks of i.p. IL7. Flow cytometry was performed on day 22 after completion of IL7 course. **B**–**F** Results of flow cytometry (*n* = 4 for RT, *n* = 5 for all other cohorts). Flow cytometry results were gated for infiltrating lymphocytes, proliferation markers, and effector function. **G** Differences in CD127 expression between Tregs, conventional CD4 T-cells, and CD8 T-cells. **H** Immunofluorescence staining verifying colocalization of CD8 and CD127. Yellow arrow showing a representative example of co-staining. **I** Bulk tumor RNA sequencing showing upregulation of chemokine release in RT treated mice (*n* = 3) three days post RT compared to untreated mice (*n* = 3). Statistics were computed using one-way ANOVA (**B**–**G**). The mean ± SEM is shown (**B–G**). *p < 0.05, **p < 0.01, ***p < 0.001, ****p < 0.0001

Given the early activation of lymphocytes, we repeated the experiment at a later timepoint to better understand the lymphocyte response in the LN and TME. We performed flow cytometry to characterize the immune changes in the TME at day 22 (Fig. 4A). We found that the combination treatment of RT + IL7 significantly stimulates a large influx of CD8 T-cells into the TME (Fig. 4B). These CD8 T-cells had heightened effector function as demonstrated by increased secretion of interferon gamma (IFN*γ*) and expression of Ki-67 (Fig. 4C, D). Given the increased CD8 T-cell influx and activation, there was a corresponding increase (*p* = 0.1047) in Type 1 conventional dendritic cell (cDC1) migration (defined as CD8^+^CD11c^+^MHCII^+^CD45^+^) (Supp Fig. 3A). No significant differences were detected in PD1 expression across different cohorts, and there was no increase in B-cell tumor infiltration (Supp Fig. 3B, C).

When examining the CD4 T-cell population, there was less notable activation. There was no difference in conventional CD4 (FoxP3^−^) T-cell influx (Fig. 4E) though there was increased CD44 expression (Supp Fig. 3D). In addition, there was no increase in the CD4 Treg (FoxP3^+^) population, which is an immunosuppressive cell phenotype associated with poor prognostic outcomes [31] (Fig. 4F). This may be explained by the low expression of IL7R on Tregs compared to effector T-cell populations. CD8 T-cells were observed to have a threefold increase in IL7R expression compared to the Treg population (Fig. 4G). Verification of CD8 and IL7R colocalization was performed using immunofluorescence (IF) staining. Visual confirmation shows high co-expression of CD8 in conjunction with IL7R (CD127) as expected based on the flow cytometry results (Fig. 4H).

To better understand how RT improved TIL concentration, RNAseq was performed on irradiated LY2 tumors and compared to an untreated group. Increased expression of multiple known chemokines associated with T-cell recruitment into the TME, including CXCR3, CXCL9 CXCL10, and CXCL13 [32–34], was observed in the RT group compared to the untreated group (Fig. 4I), suggesting that RT provides a strong chemotaxis force for lymphocytes to enter the TME.

### CD8 T-cell memory-like phenotype upregulated by IL7

**Fig. 5:**
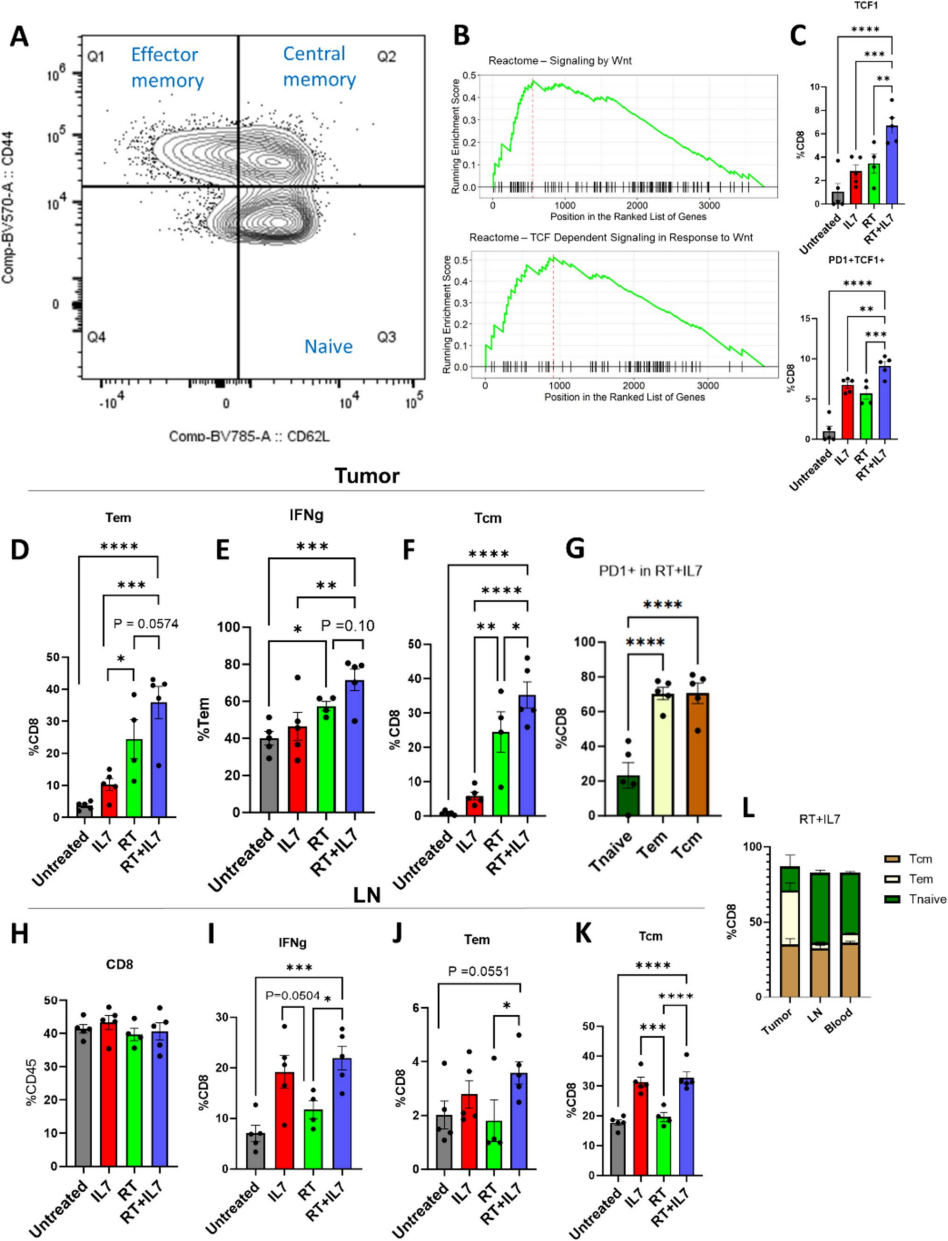
IL7 increases memory-like CD8 T-cell population in the TME and the LN. **A** Memory T-cell gating strategy for flow cytometry for CD8 + T-cells. Central memory T-cells (Tcm) defined by CD62L^+^CD44^+^, effector memory T-cells (Tem) defined by CD62L^−^CD44^+^, and naïve T-cells (Tn) defined by CD62L^+^CD44^−^. The representative gating is from CD8 T-cells in the tumor. **B** Gene set enrichment analysis (GSEA) using the Reactome pathway was performed on bulk tumor proteomics. When comparing IL7 to the untreated group, this analysis revealed increased Wnt and TCF signaling. **C** Corresponding tumor flow cytometry that examines TCF1 and PD1 expression on CD8 T-cells as a proxy for stemness. **D**–**K** Flow cytometry results focusing on CD8 T-cell memory formation in the tumor and LN (*n* = 4 for RT, *n* = 5 for all other cohorts). PD1 expression was used as a surrogate for antigen experience in (**G**). **L** Distribution of Tcm, Tem, and Tn across the tumor, LN, and blood compartments for the RT + IL7 cohort. Statistics were computed using GSEA (**B**) and one-way ANOVA (**C**–**K**). The mean ± SEM is shown (**C**–**K**). *p < 0.05, **p < 0.01, ***p < 0.001, ****p < 0.0001

Next, the relationship between IL7 and memory development was further investigated. Central memory T-cells (Tcm), effector memory T-cells (Tem), and naive T-cells (Tn) were defined using the markers CD62L and CD44 [35–37] (Fig. 5A). Tn were characterized by negative PD1 expression and low IFNγ expression (Supp Fig. 4A). Comparison of proteomics pathways using gene set enrichment analysis (GSEA) between the IL7 and untreated groups demonstrated upregulation of the Wnt/TCF signaling pathway (Fig. 5B). Flow cytometry similarly showed increased TCF1 expression on CD8 T-cells in the RT + IL7 group (Fig. 5C, representative gating in Supp Fig. 4B). We identified a subpopulation of TCF1^+^PD1^+^ CD8 T-cells that have been described as a precursor exhausted population (*T*pex) with an ability to undergo self-renewal and have high proliferative capability [38] (Fig. 5C).

In the RT + IL7 cohort, the TME had a high influx of both Tem and Tcm. Tem had high effector function as evidenced by increased IFNγ expression (Fig. 5D–F). The Tem and Tcm populations were more antigen experienced than the Tn population with increased PD1 expression (Fig. 5G). When analyzing CD8 T-cells in the LN, there was an increased IFNγ signature in the IL7 treated groups (Fig. 5H, I). Moreover, it was found that central memory T-cells predominately reside in this compartment compared to effector T-cells (Fig. 5J, K). While there is also increased Tem presence with IL7 treatment, Tcm outnumber this cell type by nearly 15-fold (Fig. 5L). Similar trends were seen in the blood, where central memory cells were more prevalent than effectors (Supp Fig. 5A–E). Overall, this shows that IL7 expands a subpopulation of memory-like CD8 T-cells.

### Lymphoid progenitors stimulated in the bone marrow

**Fig. 6:**
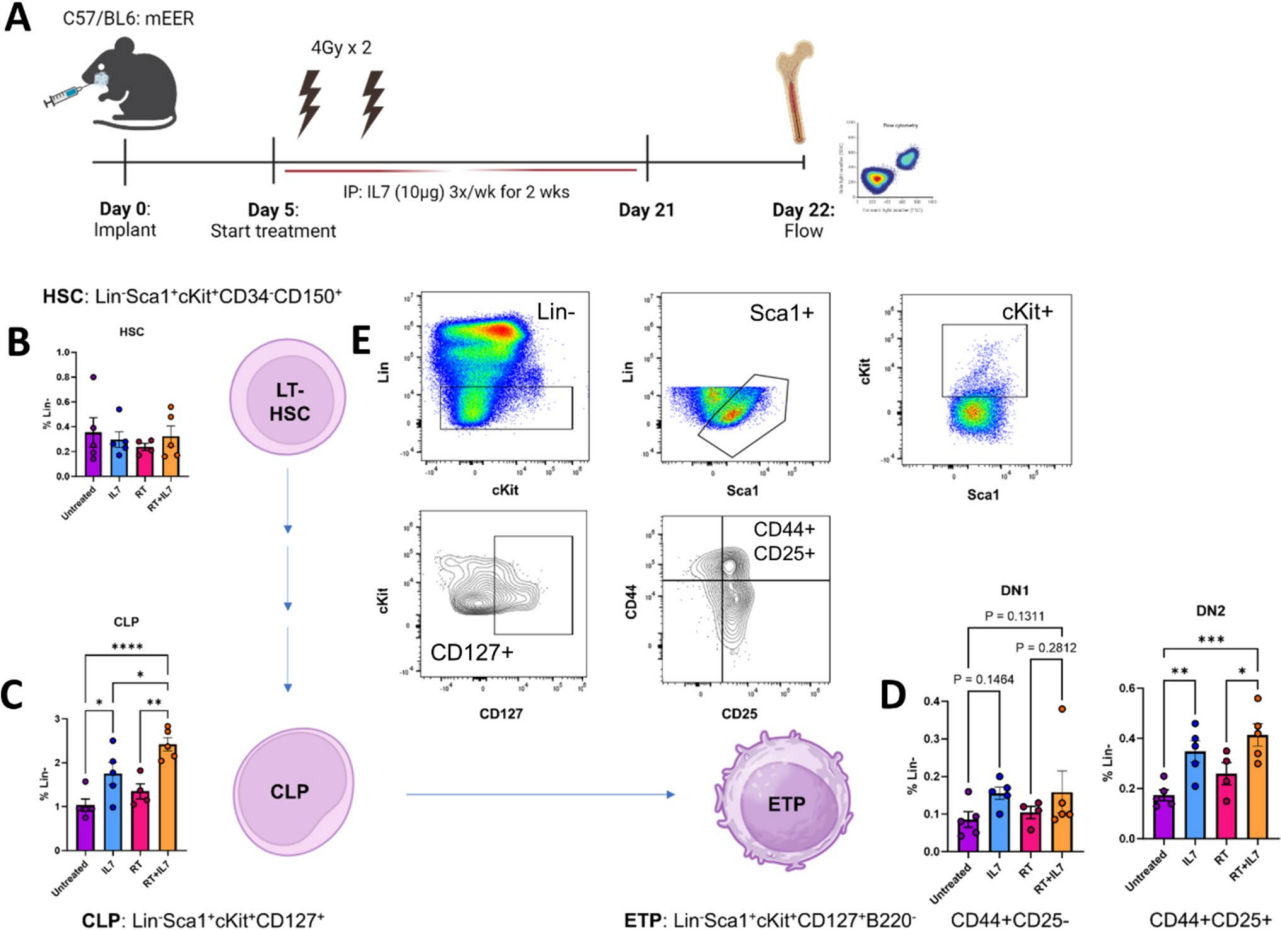
IL7 in combination with RT stimulates precursor T-cell development in the bone marrow. **A** Experimental schematic showing timing of treatments. Two doses of 4 Gy RT and two weeks of IL7 via i.p. injections were given. Bone marrow was harvested on day 22 following completion of treatment. **B**–**D** Results of bone marrow flow cytometry (*n* = 5 for each cohort) with lineage tree tracing. **E** Gating strategy used for identification of hematopoietic stem cell (HSC, Lin^−^Sca1^+^cKit^+^CD34^−^CD150^+^), common lymphoid progenitor (CLP, Lin^−^Sca1^+^cKit^+^CD127^+^), and early thymic progenitor (ETP, Lin^−^Sca1^+^cKit^+^CD127^+^B220^−^). Statistics were calculated using one-way ANOVA (**B**–**D**). The mean ± SEM is shown (B, D, and E). *p < 0.05, **p < 0.01, ***p < 0.001, ****p < 0.0001

Given reports of IL7’s effect on bone marrow stimulation [39], we investigated the effect of IL7 on the lymphoid progenitor pathway. Bone marrow was harvested on day 22 and used for flow cytometry with a specific gating strategy designed for precursor T-cells (Fig. 6A, E). No difference was seen across all 4 groups in the concentration of the long-term hematopoietic stem cell (LT-HSC) (Fig. 6B). However, with IL7 treatment, there was a significant increase in downstream common lymphoid progenitor cells (CLPs) (Fig. 6C) and early thymic progenitor cells (ETPs). Specifically, there was an increase in double negative 2 (DN2) T-cells (*p* < 0.001) and double negative 1 (DN1) T-cells (*p* = 0.1311) (Fig. 6D). In summary, our data show a secondary method through which IL7 can exert its antitumor effect by stimulating precursor lymphocyte proliferation.

### Treg depletion augments IL7 mediated antitumor response

**Fig. 7:**
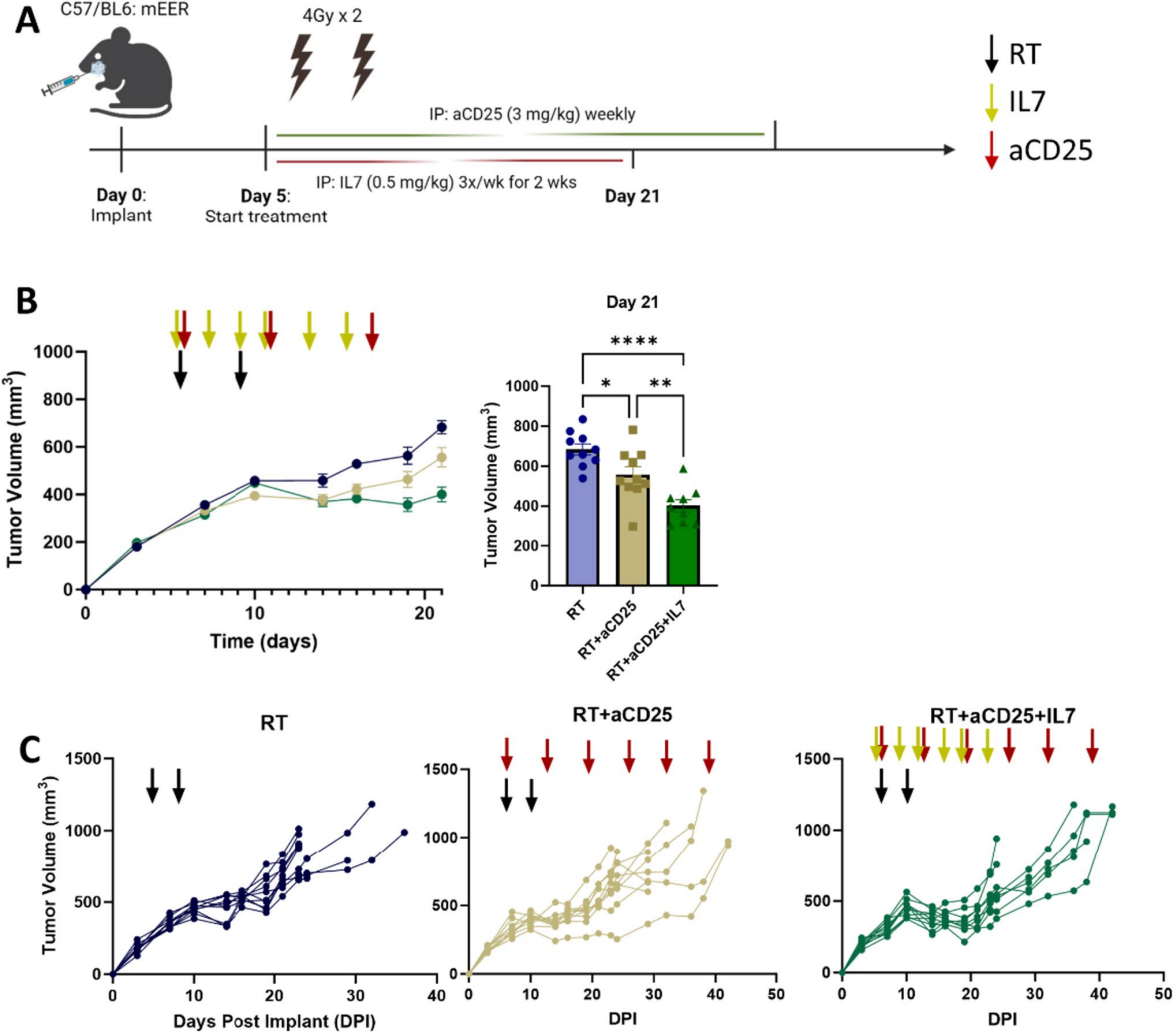
Treg depletion augments IL7 mediated tumor growth delay. **A** Experimental schematic with timing of RT, aCD25, and IL7 injections. RT was administered in two 4 Gy fractions. IL7 was administered three times a week via i.p. injection for 2 weeks, and aCD25 was given via i.p. injection once per week. **B** Tumor growth curves for each treatment group (*n* = 10 for each group). Black arrow indicates RT administration; yellow arrow indicates IL7 injection; red arrow indicates aCD25 injection. **C** Individual tumor growth curves for all cohorts. Statistics were calculated using one-way ANOVA (**B**). The mean ± SEM is shown (**B**). *p < 0.05, **p < 0.01, ***p < 0.001, ****p < 0.0001

Given the robust CD8 T-cell memory response, but without any meaningful effect on CD4 T-cells or Tregs, we reasoned that the efficacy of IL7 would be improved by targeting the Treg axis, a well-documented driver of therapeutic resistance in HSNCC [30, 31, 40]. To test this, Tregs were pharmacologically depleted using a previously validated anti-CD25 antibody (αCD25) [30, 31, 41, 42]. Mice were divided into 3 groups: RT, RT + αCD25, RT + αCD25 + IL7 (Fig. 7A). IL7 was found to work in synergy with αCD25 to augment antitumor effects (Fig. 7B–C), demonstrating that these two drugs may be used synergistically for controlling tumor burden.

## Discussion

FDA approved immunotherapies for HNSCC have centered around manipulating the PD1/PD-L1 axis [43, 44] and historically have been limited to the setting of metastatic and recurrent disease [45, 46]. Clinical trials to date using immunotherapy for locally advanced HNSCC have either demonstrated no significant improvement in overall survival (NCT02952586) or are still in early phases [14, 16]. Consequently, there is a need to develop and evaluate new immunotherapy strategies for management of early stage and locally advanced disease. This work provides a preclinical rationale for the use of IL7 as a potential immunotherapeutic agent that is outside the conventional ICB pathway. Our data show that IL7 in conjunction with RT can be used to control tumor growth in both HPV+ and HPV− preclinical models of HNSCC.

IL7 is critical for lymphocyte development and maintenance, and knockout of the IL7R gene leads to a dearth of lymphocyte activity [47]. While IL7 has multiple roles along the T-cell developmental pathway, T-cell survival and maintenance in particular are regulated by IL7 secretion [21, 24, 48]. Recent research in chimeric antigen receptor (CAR) T-cell engineering has taken advantage of this property to create CAR T-cells that constitutively express IL7. These CAR T-cells have been shown to survive longer and have increased antitumor activity [49–51]. Similarly, IL7 was found to increase proliferation of circulating CD4 and CD8 T-cells leading to reduced flank tumor growth in a hepatocellular carcinoma model [52]. Like our results, that study showed the addition of RT allowed for substantially increased tumor reduction compared to IL7 alone.

In addition to survival and proliferation, IL7 is known to mediate the development and maintenance of memory T-cells. Usually after a T-cell has encountered its cognate antigen and cleared the pathogenic invasion, it will undergo apoptosis. However, a small proportion will differentiate into memory T-cells [53]. Several studies have shown that IL7 can drive the differentiation of naive T-cells into a memory phenotype, specifically in infections such as Leishmaniasis and tuberculosis [54, 55]. Our study shows IL7 also increases central and effector memory T-cells in cancer immunity, with the T-cell response being compartment specific. The effector T-cell response was contained to the TME, whereas the central memory T-cells were housed in the draining LN.

TCF1 is a T-cell specific transcription factor that plays an important role in self-renewal [56]. TCF1 was seen to be upregulated by CD8 T-cells in the TME in our experiments. GSEA demonstrated upregulation of the Wnt and TCF1 signaling pathways in the IL7 treated cohorts. Biologically, TCF1 lies downstream of the Wnt/*β*-catenin signaling pathway [57] and plays a role in memory formation [58–60]. Recent studies in tumor immunology have identified a population of T-cells expressing PD1 and TCF1 that exhibit a progenitor exhausted phenotype with high self-renewal and proliferative capacity [61, 62]. These cells can be stimulated to expand with ICB and differentiate into effector populations associated with improved outcomes in human cancer [63, 64]. Our study shows that IL7 administration upregulates TCF1 signaling which may be driving T-cells into this stem-like state.

The cellular expression of the IL7 receptor provides key insight into the pharmaceutical targets of IL7 agonism. Our flow cytometry results indicate that within the TME, CD8 T-cells have severalfold higher expression of IL7R compared to immunosuppressive Tregs, consistent with existing literature [25]. There is mixed data on the role of IL7R on Tregs. Some studies have indicated that IL7 may play an important role in the Treg developmental phase [65, 66], whereas others have found that Tregs in secondary lymphoid organs do not rely on IL7 for their homeostatic maintenance [67]. We detected low expression of IL7R by Tregs in the TME, allowing for selective activation of CD8 T-cells. One mechanism of Treg immunosuppression is IL2 sequestration via expression of the high affinity CD25 surface receptor. IL2 drives proliferation of a highly cytotoxic T-cell subpopulation that express KLRK1 and IL7R on their cell surface [68]. Subsequently, we hypothesized that adding CD25 depletion to our IL7 agonist would further dampen tumor growth, which indeed was observed. These data demonstrate an effective immunotherapy strategy of combining effector T-cell agonism with Treg depletion for future drug design.

Prior literature has indicated that IL7 is crucial for lymphocyte development in the bone marrow [69]. In particular, CLPs are known to express CD127 on their cell surfaces [70]. Our experiments show that IL7 and RT + IL7 administration increases the number of T-cell precursors. With this in mind, we propose that IL7 has a two-pronged approach to invigorating the immune response. First, IL7 promotes CD8 T-cell influx and effector function in the TME, and second, IL7 stimulates lymphocyte proliferation via the CLP/ETP differentiation pathway. Total body irradiation has been linked to bone marrow suppression [71, 72], spurring the development of focal RT regimens to reduce hematologic toxicity [73]. Focal irradiation with limited bone marrow involvement appears to have mild effects on circulating white blood cells, and local areas of bone marrow injury can regenerate with time [74, 75]. Local RT has also been shown to recruit bone marrow precursors to the irradiated site [76]. One corollary to our study would be an alternative use of IL7 as a treatment for lymphopenia, whether it be in the setting of radiation induced lymphopenia or other general leukopenia disorders.

Examining the differences between tumor growth in the IL7 alone group compared to the RT + IL7 group provides insight into immunotherapy design. When examining the draining LNs, the IL7 group had similar proliferation of CD8 T-cells as the RT + IL7 group. However, when analyzing the TME, the IL7 group had substantially less TILs. Presumably this is from a lack of a strong chemo-attractive force to drive lymphocytes into the TME. Our RNAseq data demonstrates that RT increases the release of numerous cytokines, which are otherwise not abundantly present in the IL7 group. This may be important for future immunotherapy design, where not only must immune cell populations become activated, but they also must be stimulated to migrate into the tumor. Such a treatment paradigm has been recognized and implemented in CAR T-cell research, where CAR T-cells are being engineered to express both a stimulant (such as IL7) as well as a chemokine (such as CCL19 or CCL21) [77–81].

In summary, our data show that IL7 in combination with radiotherapy provides a novel treatment regimen that can control tumor growth in HNSCC preclinical models. We find that this immunotherapy strategy stimulates the influx of CD8 T-cells into the TME, increases the proliferation of precursor T-cells in the bone marrow, and its effect can be augmented with Treg inhibition. Our results identify IL7 as a potential new translational therapy that adds to the existing armamentarium of immunotherapeutic agents.

### Supplementary Information

Below is the link to the electronic supplementary material.

Supplementary file1 (DOCX 1803 kb)
