# Supplementary material for: IL7 in combination with radiotherapy stimulates a memory T-cell response to improve outcomes in HNSCC models
Source: Cancer Immunol Immunother. 2024 Mar 30;73(5):90. doi: 10.1007/s00262-024-03664-y (PMC10981637; doi:10.1007/s00262-024-03664-y)
Supplement: Supplementary file 1 — Supplementary file1 (DOCX 1803 kb) [file 262_2024_3664_MOESM1_ESM.docx]

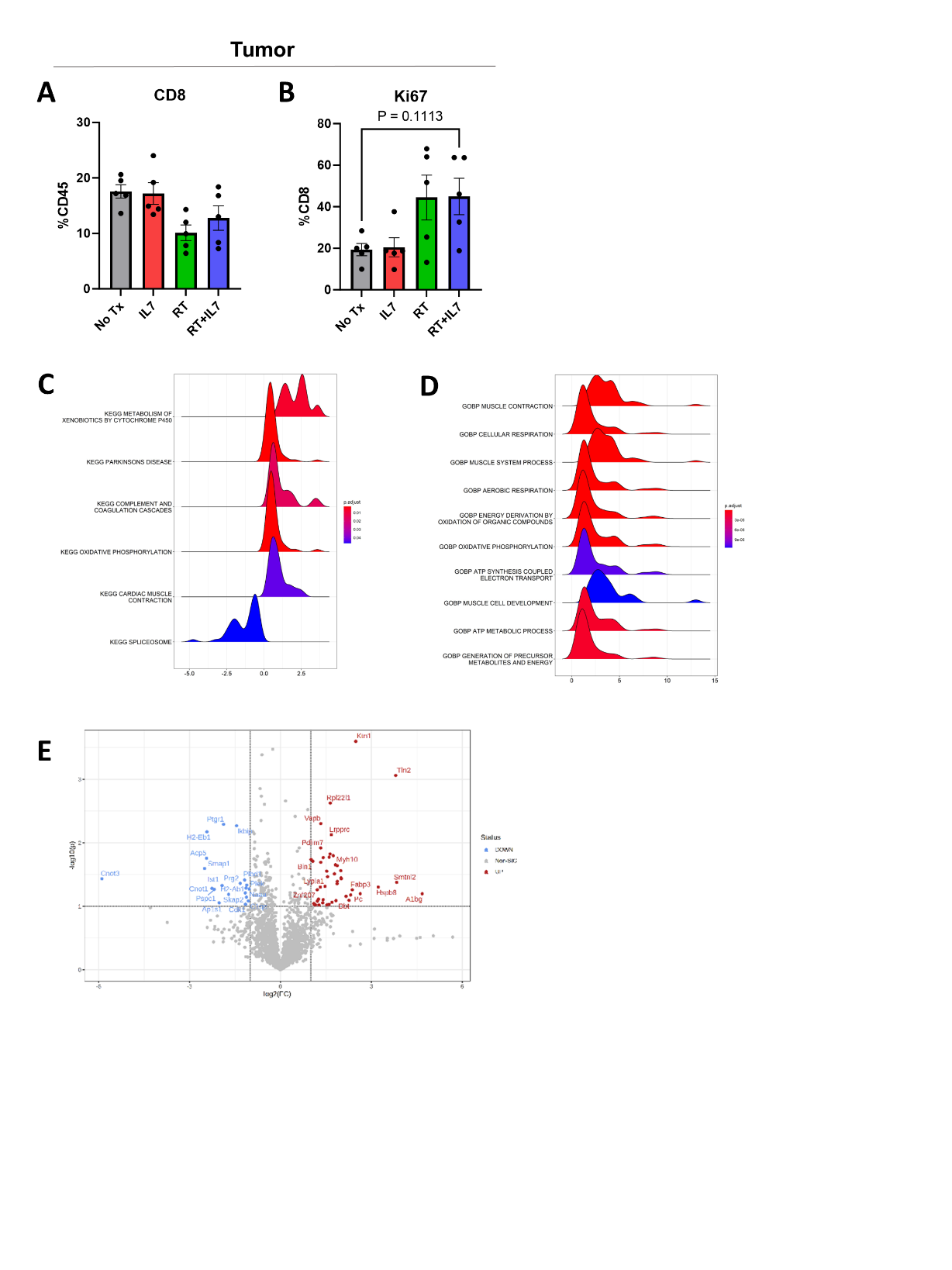


**Supplemental Figure 1.**

(A-B) Early timepoint flow cytometry results at day 15 post implantation performed on the tumor. Average CD8 T-cell infiltration (A) and Ki67 expression (B) on CD8 T-cells is shown. Data is shown as the mean ± SEM. One-way ANOVA testing was used to compute p-values. N=5 for each group.

(C-D) Bulk tumor proteomics data from day 15 post implantation. KEGG pathway analysis (C) shows changes in metabolic pathways that are upregulated in RT+IL7 compared to RT group. Major pathways upregulate include metabolism by cytochrome P450, complement and coagulation cascades, and oxidative phosphorylation. GO Biologic Processes pathway analysis (D) similarly finds increased metabolic processes in the bulk tumor proteomics data with hits along the cellular respiration, aerobic respiration, oxidative phosphorylation, and ATP synthesis coupled electron transport pathways.

(E) Volcano plot showing differentially expressed proteins in the tumor between the RT+IL7 and RT cohorts.


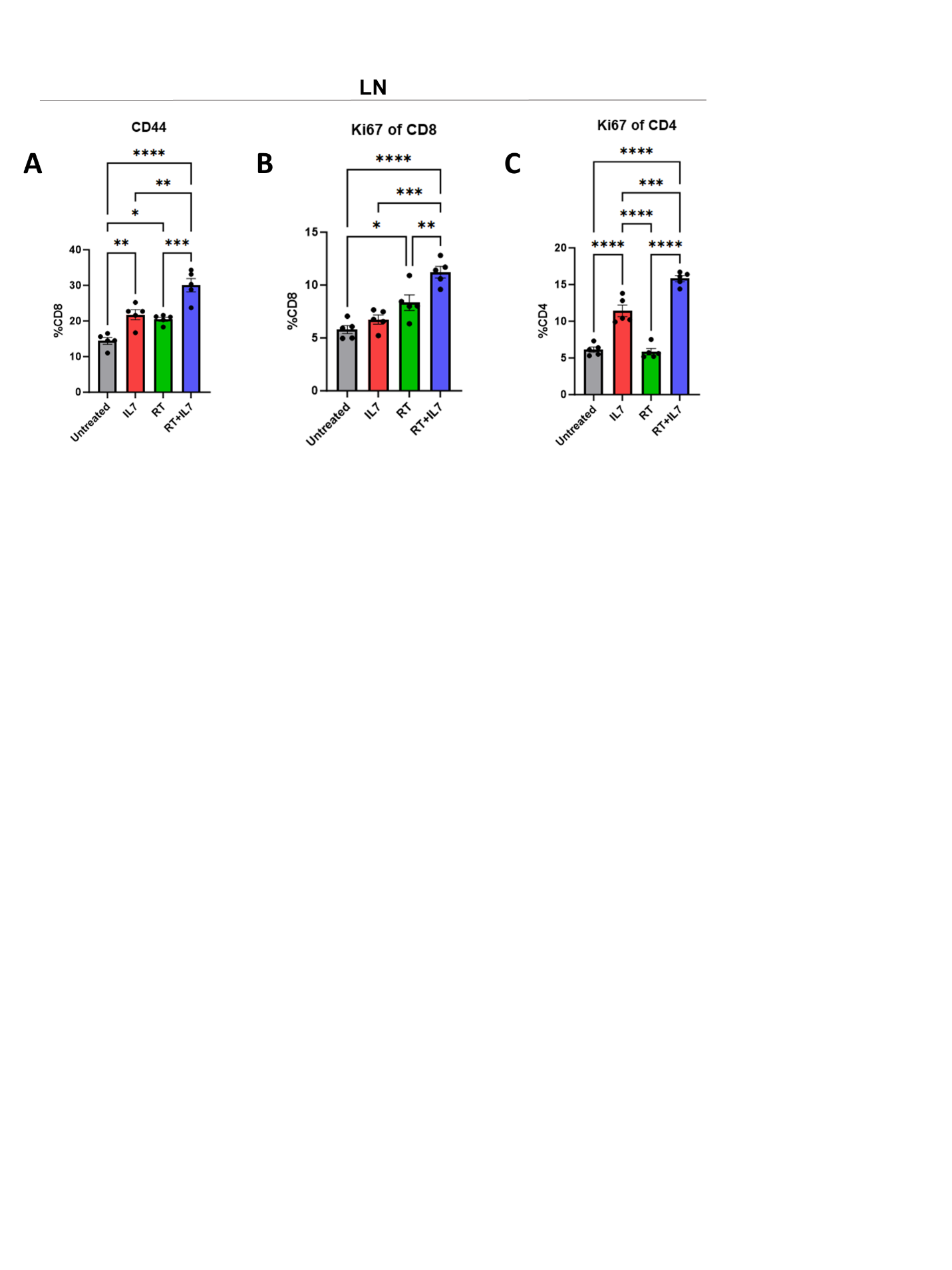


**Supplemental Figure 2.**

(A-C) Early timepoint flow cytometry of the tumor draining lymph node (n=5 for each group). CD44 and Ki67 were used as surrogate markers of lymphocyte activation. Bar chart showing the mean ± SEM with p-values calculated by one-way ANOVA testing. *p<0.05, **p<0.01, ***p<0.001, ****p<0.0001.


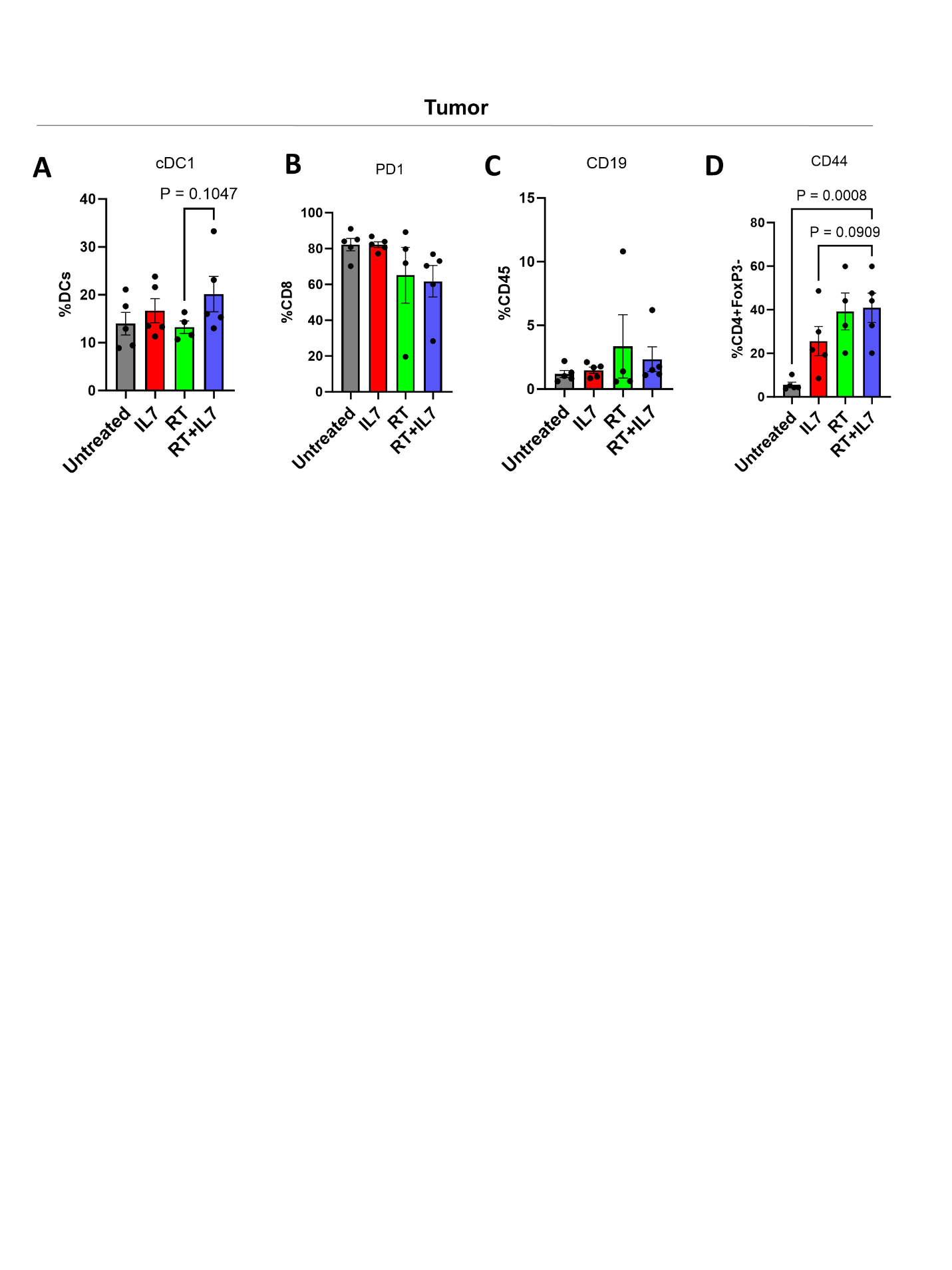


**Supplemental Figure 3:**

(A-D) Late timepoint flow cytometry results of the tumor (n=4 for RT, n=5 for all other groups). Data showing gating for tumor infiltrating cDC1s (CD8^+^MHCII^+^CD11c^+^CD45^+^), B-cells (CD19^+^CD45^+^), PD1 expression on CD8 T-cells, and CD44 expression on conventional CD4 T-cells. Bar chart showing mean ± SEM with p-values calculated by one-way ANOVA testing.


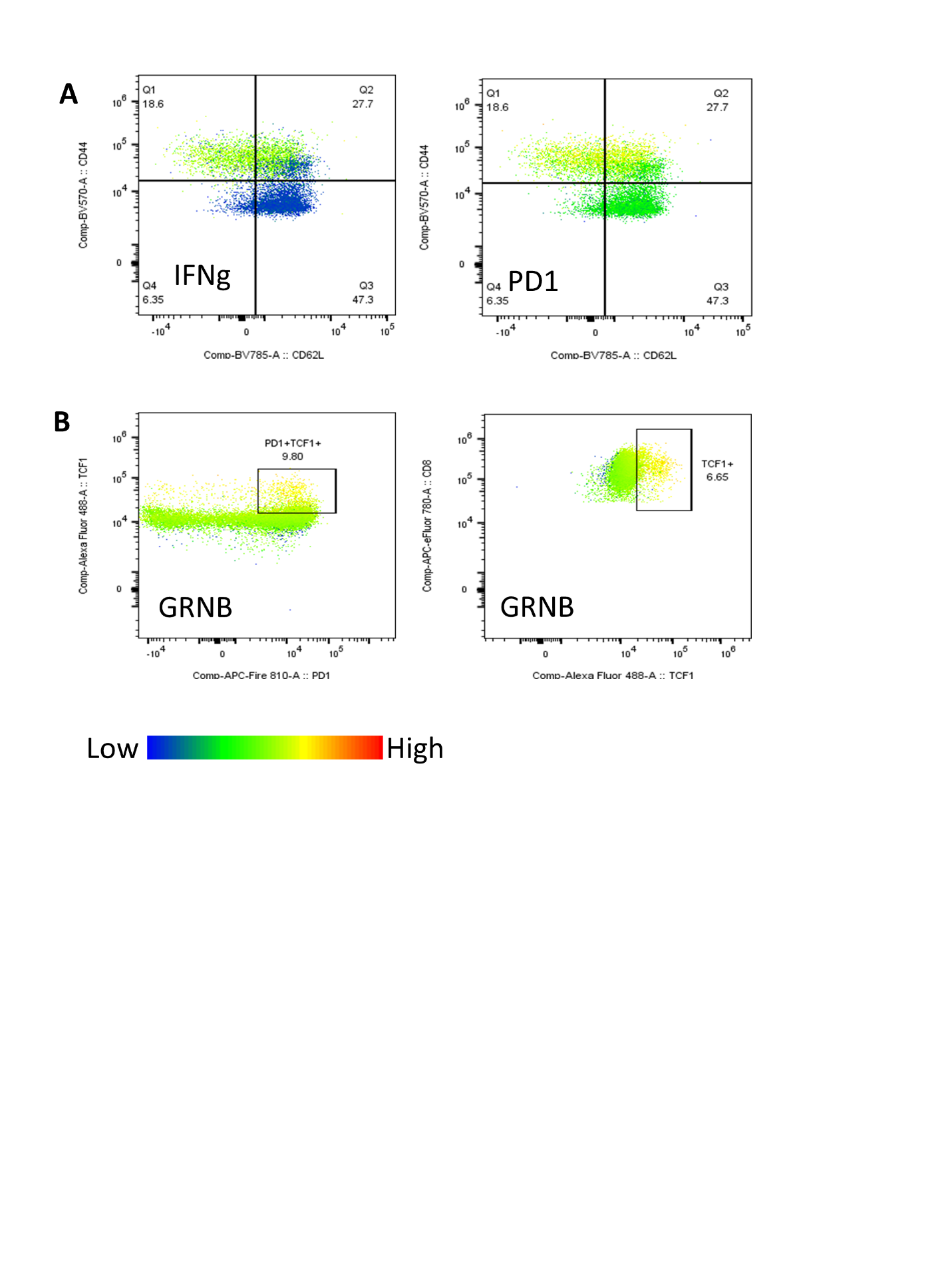


**Supplemental Figure 4:**

(A) Representative gating strategy for memory phenotype using CD44 and CD62L. CD44 and CD62L were gated into quadrants. Expression of IFNγ and PD1 were used as proxies to assess activation status of lymphocytes.

(B) Representative gating strategy for TCF1 and PD1. Granzyme B (GRNB) overlay to assess cytotoxic function.

**
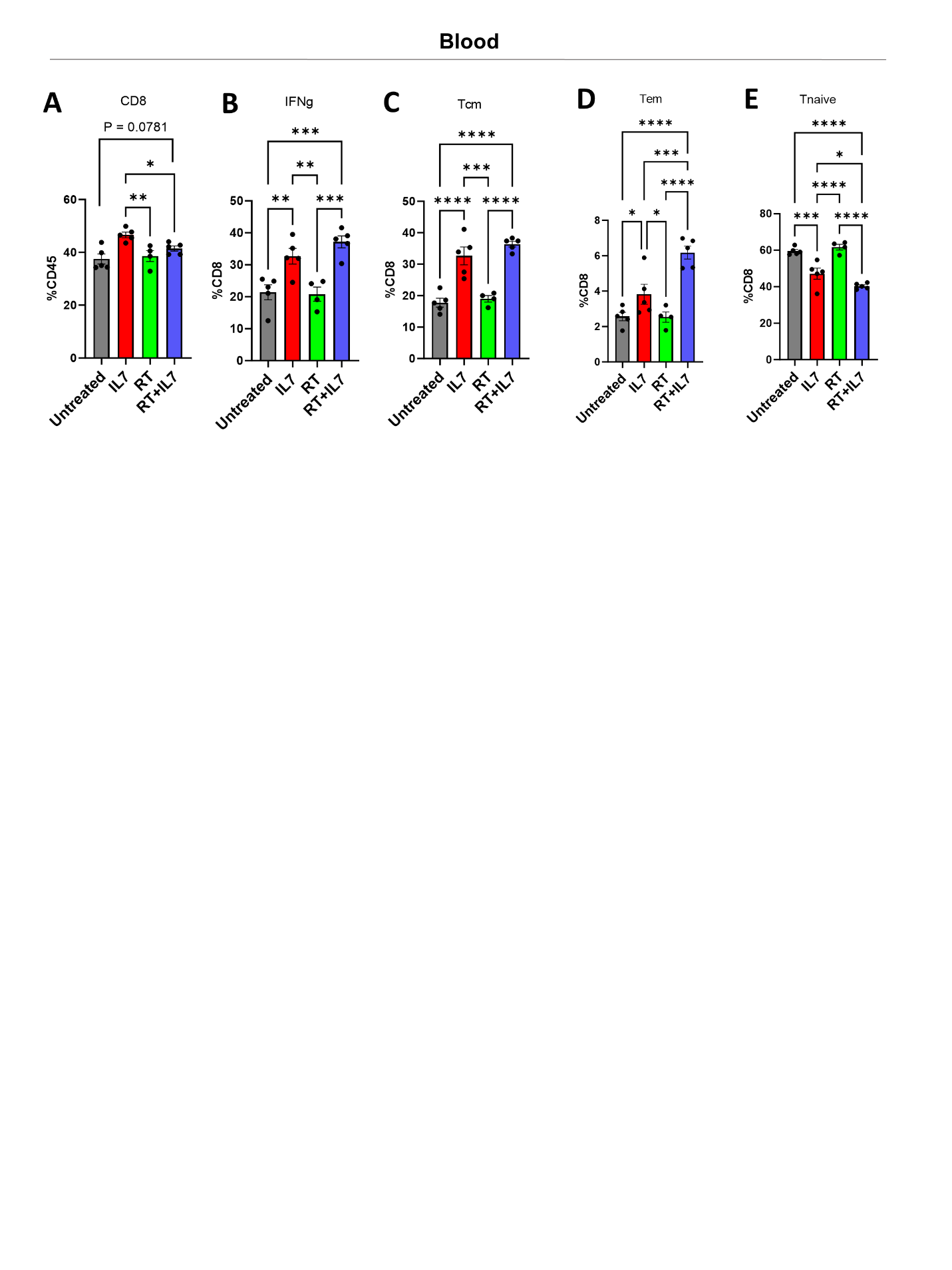
**

**Supplemental Figure 5:**

(A-E) Day 22 flow cytometry results of lymphocytes in the blood (n=4 for RT, n=5 for all other groups). CD8 T-cell influx, activity, and memory markers were assessed. The mean ± SEM are shown as bar charts for each cohort. Statistics computed through one-way ANOVA testing. *p<0.05, **p<0.01, ***p<0.001, ****p<0.0001.
